# Ray Amputation in a Traumatic Diabetic Foot

**DOI:** 10.7759/cureus.24444

**Published:** 2022-04-24

**Authors:** Rebecca Lawrence, R. Xenophon Kirby, Anderson E Ikeokwu

**Affiliations:** 1 General Surgery, May Pen Hospital, May Pen, JAM

**Keywords:** uncontrolled diabetes, diabetic foot classification system, diabetic foot scoring metric, albumin infusion, polymicrobial bacteremia, digit amputation, vinegar, jamaica, diabetic foot, ray amputation

## Abstract

Diabetic foot is a complex syndrome that is co-morbid with other diabetic complications such as peripheral arterial disease (PAD) and peripheral neuropathy. Patients with the diabetic foot are increasingly prone to diabetic foot ulcers (DFUs) due to a high infection susceptibility and poor wound healing possibly prompting the need for minor or major amputations. We herein highlight the case of a 47-year-old male with a traumatic diabetic foot who necessitated a Ray amputation (RA). The notable aspects of this case are the late presentation of a patient with uncontrolled diabetes who could have avoided this complication if he went to seek help earlier and how diabetic foot is controlled and managed in a low-income resource setting. This case also highlights how physicians can better diagnose and treat diabetic foot complications with a scoring metric.

## Introduction

Diabetes mellitus (DM) is a metabolic condition that poses an epidemic in the country of Jamaica where the condition is responsible for a heavy burden of chronic illness, long-term disability, and premature loss of life throughout the Caribbean [1]. Approximately 11.9% or 236,200 Jamaicans, 15 years and older have diabetes. The prevalence increases with age and more women compared to men have diabetes [2]. There are 92.5% of Jamaicans 15 years and older with diabetes that are on treatment. Of those on treatment, only 27.5% are controlled [2].

In a country where over 70% of diabetics are poorly managed, there are innumerable risks, as diabetic patients are immunocompromised. In a patient with impaired glucose control or chronic hyperglycemia, these concerns include increased susceptibility to infections due to decreased complement response and the dysfunctional activity of leukocytes.

Leukocyte recruitment results in the production of leukotrienes and reactive oxygen species, and the expression of pro-inflammatory cytokines ultimately leading to impaired host defense and decreased bactericidal activity [3]. Chronic hyperglycemia can cause an acidotic state, lack of oxygen perfusion, and decreased blood flow thereby compromising the skin which increases susceptibility to skin and soft tissue infections (SSTI) in events of trauma. As a result of poor perfusion and capillary damage, irreversible neuropathy develops with time, and patients are unlikely to be observant when trauma occurs until an infection sets in place.

Diabetic foot is a complex syndrome that is co-morbid with other diabetic complications such as peripheral arterial disease (PAD) and peripheral neuropathy. Patients with the diabetic foot are increasingly prone to diabetic foot ulcers (DFUs) due to a high infection susceptibility and poor wound healing possibly prompting the need for minor or major amputations. In patients with any forefoot necrosis or infections, minor amputations such as digit, ray, and transmetatarsal amputations have been demonstrated to be the most beneficial with limb salvage rates and maintaining foot and ankle biomechanics [4]. Revascularization, anti-infection treatment, surgical operations, and postoperative wound care are the current interventions undertaken in modern clinical practice [4]. The patient as reported in this case has a traumatic wound in his diabetic foot, which has caused wet gangrene of his toe, necessitating a Ray amputation (RA).

## Case presentation

A 47-year-old male with a known history of DM presented to the Accident and Emergency Department with a referral from a private practitioner for an infected left second toe. The patient explained that he suffered a cut on his foot from a sharp wooden object that he did not initially feel whilst walking barefoot over a month ago. He did not seek any treatment for the same as he is afraid of hospitals. In the following days since the cut, his toe started getting swollen and erythematous. As the wound progressed over the weeks to come, he noticed blackening of his toe, swelling up to his ankle, pain in the ankle, and malodorous, bloody purulent exudate from the site of the injury. The patient then presented to his private practitioner two days ago who referred him to our institution for further management.

The patient has had DM for over 10 years and was maintained on metformin, however, he has been non-compliant with his medication for one year. He had debridement of an infected cut on the right foot’s plantar surface at our institution in 2019. The patient has a family history of DM, his grandfather had DM, and his 24-year-old son is also a newly diagnosed diabetic. The patient has no known drug allergies and currently uses no prescribed, over-the-counter or herbal medications. He is a Mortician by profession and lives alone in a flat. The patient admits to no tobacco smoking and use of illicit drugs; however, he drinks alcohol socially and is CAGE positive for alcoholism which is a substance abuse screening tool. There is no recent travel history. He has been able to ambulate but continues to have ankle and knee pain. He has had a fever continued for over five days, diarrhea which resolved a week ago, occasional palpitations for over two months, and admits to no cough, shortness of breath, or chest pain.

The patient’s vitals recorded on arrival included a temperature of 100.4 degrees Fahrenheit, respiratory rate of 20 breaths per minute, pulse of 118 beats per minute, blood pressure (BP) of 158/90 mmHg, and glucose meter reading (GMR) of 23.4 mmol/L. On examination of his left lower limb, the skin was erythematous, edematous, and tender to touch up to the distal one-third of the left leg. The second toe of the foot was black necrotic with mild purulent and malodorous discharge and no sensation. Using a 128-Hz tuning fork, absent vibration sensations were noted at the interphalangeal (IP) and metatarsophalangeal (MTP) joints, but present at the ankle joint and knee joint. The patient has decreased pinprick sensations on the plantar surface of the left foot. There was also decreased range of motion in the left leg due to edema and an ankle-jerk reflex could not be performed due to ankle pain experienced by the patient at the time. There were no superficial vein dilations or ulcers. There was a diminished dorsalis pedis and posterior tibial pulse but the popliteal and femoral pulses were intact. The right lower limb is mostly devoid of hair with small darkened discolorations above the ankle. Vibration sense is identified at interphalangeal (IP) and MTP joints with decreased pinprick sensations on the plantar surface of the right foot. A well-healed scar was noted on the plantar surface of the foot. All pulses were intact with an adequate range of motion.

After admission to the male surgical ward, blood was drawn from the patient for a complete blood count (CBC), urea and electrolytes (U&E), and a blood group and cross-match. The patient had an anion gap of 13.5, serum osmolality of 286, and hemoglobin of 9.9 g/dL. The blood group and cross-match showed the patient had an O-negative blood type and due to his low hemoglobin levels, he consented to a blood transfusion.

After a thorough explanation of the surgical procedure, indications, and complications, the patient gave us informed consent to perform a RA and extensive debridement. He was scheduled to have surgery the next day, as a part of the pre-operative assessment; he had an electrocardiogram (ECG) and chest X-ray (CXR) done for the anesthetic team to clear him for surgery. The patient had a foot X-ray done to assess any signs of Charcot’s Foot or osteomyelitis which were ruled out. The patient got a wound swab done before sterilization of the foot which would have occurred during surgery and to guide appropriate organism-specific antibiotic treatment. Given the current climate of the global pandemic, the patient also got a COVID-19 swab. He also planned to have GMR profiling and his vitals were monitored every four hours up until his surgery.

Based on the laboratory results, the patient was resuscitated with 3 L of 0.9% normal saline intravenously and this was alternated with lactated ringers for a 24-hour period.

To be eligible for surgery, he had to have his diabetes controlled, therefore, he was given a sliding scale therapy of soluble insulin every four hours and pre-operative antibiotics: augmentin 1.2 g intravenous (IV) thrice a day, Flagyl 500 mg IV thrice a day, and panadol 1 g per os (PO) once a day. He was started on a DM diet.

**Figure 1:**
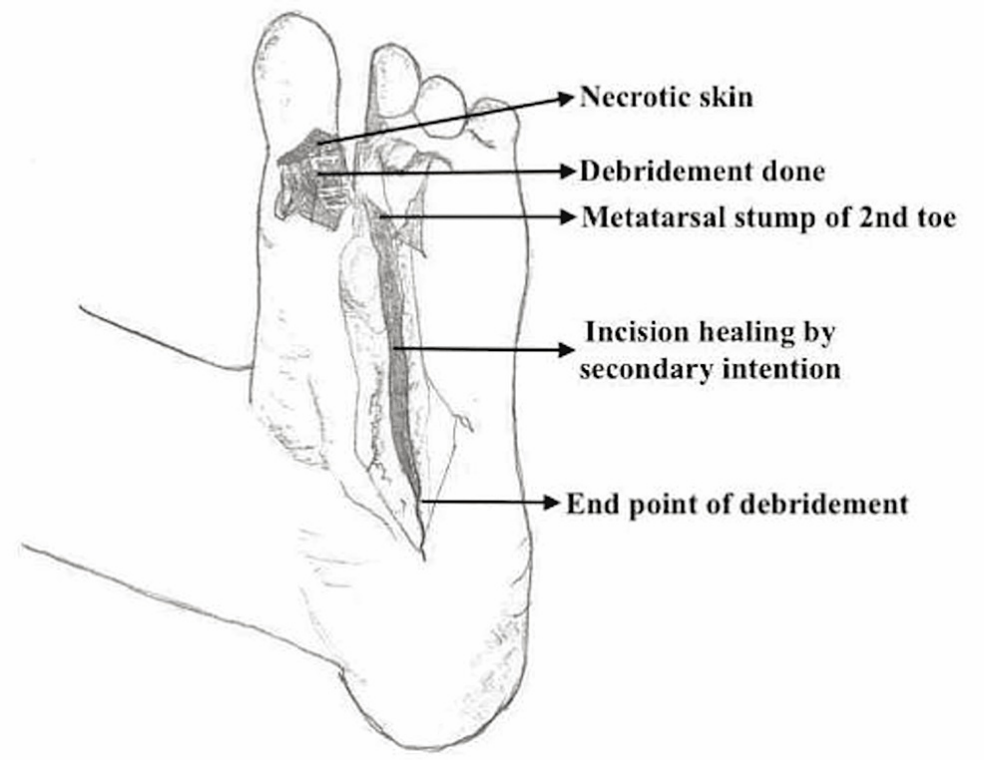
Labeled illustration of post-operative foot of the patient.

At surgery, an ankle block was administered as an anesthetic. A cake-slice incision was made to have the RA done on the second left toe leaving a metatarsal stump. Following this, the incision site was lengthened due to the presence of extensive necrotic tissue. The necrotic tissue was debrided and the wound was irrigated with 0.9% normal saline. The wound was packed and dressed accordingly and is expected to heal by itself with granulation tissue causing closure, which is also referred to as secondary intention usually requiring dressing changes up to six weeks (Figure 1).

As a part of the patient’s post-operative management, post-operative day 1, the patient was given the following analgesic medications: Baralgin 1 g IV thrice a day, Pethidine 75 g intramuscular (IM), and Gravol 50 mg IM every eight hours for the next two days. He recommenced Metformin 1 g per oral twice a day and Reclide 30 mg per oral once a day. It was imperative to continue daily dressing changes of the wound with a saline wash. Since the wound culture results were not available as of yet, Microdacyn spray and antibiotics namely augmentin 1.2 g IV and Flagyl 500 mg IV thrice a day were started. On post-operative day 5, during the daily dressing changes at ward rounds, the patient’s left great toe was ischemic with small amounts of necrotic residue and surrounding pink granulation tissue. These findings now warranted a RA of the left great toe. On post-operative day 6, the patient was given heparin 5000 units three times a day for deep vein thrombosis (DVT) prophylaxis. The wound swab results became available from the private lab and they are as follows (Table 1):

**Table 1:** Wound swab culture results.

Name of the organism	Sensitive to:	Resistant to:
Pseudomonas aeruginosa	Day 1: Zosyn, Ciprofloxacin, Gentamycin; Day 2: Imipenem, Ciprofloxacin, Gentamycin Day 3: Gentamycin, Zosyn, Ciprofloxacin, Imipenem, Meropenem	Day 1: Cefazolin
Non-Hemolytic *Streptococcus*	Day 1: Vancomycin; Day 2: Ampicillin, Augmentin, Penicillin; Day 3: Ampicillin, Augmentin, Cefaclor, Penicillin	Day 1: Ampicillin, Augmentin, Bactrim, Cefazolin, Ciprofloxacin, Penicillin, Tetracycline; Day 3: Bactrim, Tetracycline
Candida species		

With reference to Table 1, three different calls were made to the private laboratory as the initial call for wound culture results to guide the antibiotic treatment of the patient. The initial culture report was ready a week after the wound swab was sent. The wound culture was continued to be observed over a period of seven days to know if any of the organisms mentioned in the initial report developed resistance to the drugs.

The patient’s diabetes was uncontrolled up until post-operative day 10 where at this stage it was moderately controlled. On the same day, he consented to a RA of his left great toe. Due to continued tachycardia since admission, on post-operative day 14, the patient was scheduled for another ECG. The ECG findings indicated atrial flutter with a ventricular rate of 166 beats per minute. The patient was then consulted by the Internal Medicine department for a review. The patient was now given digoxin 0.25 mg per oral immediately and continued with digoxin 0.125 mg once a day. He was also referred to get a troponin I laboratory test and an echocardiogram.

By post-operative day 15, the patient’s diabetes was completely controlled and the troponin I was negative. On post-operative day 22, the patient’s wound was inspected once again to show pink granulation tissue and minimal slough. He was discharged with controlled diabetes and appropriate counseling from the Internist about his foot-care plan cognizant of any occupational hazards and medication compliance, the dietician about adapting a DM diet at home, and the following medications: metformin 1 g, carvedilol 3.25 g, and panadol 1 g. He was also given a referral to the Cardiology department. The patient was advised not to perform daily dressings by himself as he did not have a trained in-home nurse and was given a referral to the hospital's health center for dressings. He was instructed to present to ward review a week later for wound inspection and dressing changes. At the first ward review, the slough was noted at the lateral aspect of the wound. Thereby, under sterile conditions, an ankle block of 2% lidocaine diluted with 10 mL of 0.9% normal saline was given. The wound was cleaned, debrided, and dressed accordingly. At the second ward review, healthy pink granulation tissue was noted with no drainage and his follow-up appointment at the surgical outpatient department was scheduled for six months’ time.

## Discussion

Diabetic foot ulcers are more common in male diabetic patients than female diabetic patients, with a median age of 62 years old, on an average 11-year history of diabetes, 30% of whom are smokers, and 64% having co-morbidities such as hypertension and diabetic retinopathy [5]. As a male diabetic with a 10-year history of diabetes, the patient roughly matches two of the above characteristics, also under the category of the age of onset of DFUs which is 45 years of age and above. His non-healing ulcer, on the other hand, is traumatic in nature, and he tried to self-manage the wound for four weeks before seeking medical care because he was afraid of going to the hospital. However, his wound recovery was not progressing, and his condition was deteriorating, causing him to seek medical help.

In the diagnosis of a traumatic DFU with complications, a clinical judgment was made to guide the treatment and management of the condition. This patient had an initial clinical presentation of wet gangrene of the toe, surrounding necrotic tissue, and associated cellulitis warranting that the patient went through extensive debridement and a RA for the purposes of infection control and removal of dead and infected tissue.

To aid in the diagnosis of DFUs, there are various scoring metrics or assessment systems available that help provides a promptly accurate diagnosis and guide treatment. These systems are namely the Wagner’s Classification; University of Texas (UT) Classification; PEDIS (Perfusion, Extent, Depth, Infection, and Sensation) grading classification system; and DIRECT wound coding system [6]. Amongst all, the Wagner classification system was the first one to categorize DFU. It does so by assessing the depth of the ulcer and the presence of osteomyelitis or gangrene and divides the ulcers into six grades. However straightforward it might seem in a wide application, it does not consider PAD and infection for the first three grades [7]. The University of Texas system consists of grades and stages. Grades of ulcers are based on their depth and the stages are determined by the presence or absence of infection and ischemia [7]. This helps predict outcomes because infection and ischemia are included in the evaluation. However, neither system accounts for necrotic status, size of the ulcer, or vascular status [6].

As a result, none of these classification systems have been internationally recognized to guide the standard of care. To date, the DIRECT (Debridement of necrosis, Infection control, Revascularization, Exudate control, Chronicity, and Top surface) Classification System comprises various pathophysiological components of wound healing and shows a higher association with the prediction of amputation or complete healing compared to the Wagner and the UT wound classification systems [6].

Therefore, both wound experts and newer medical trainees would benefit from using a Classification system such as the DIRECT wound coding system in multifaceted cases of diabetic wounds as it would suggest the most practical treatment, simplify tracking progression or regression of wound healing, and thereby practitioners would have more accurate outcome predictions which included either revascularization, limb salvage, or amputation [6].

Diabetic foot surgeries can be classified into minor or major amputations. A minor amputation as in this patient’s case is done either at the level of the forefoot, midfoot, or hindfoot where the tibial weight-bearing stump can be preserved. Whereas, a major amputation is either done trans-tibial/below the knee, through the knee/Gritti stokes, trans-femoral/above the knee, or as a hip disarticulation. Minor amputations have significantly decreased mortality risks compared to major amputations (hazard rate 1.6 times higher for major > minor) and in two-thirds of the population, limb salvage is achieved [8].

A RA involves the excision of the toe and part of the metatarsal leaving a stump. In the case of this patient, a RA was decided based on the presence of wet gangrene, no sensation, no palpable pulse, and the presence of surrounding necrotic tissue. Other indications for a RA include dry gangrene of a toe, osteomyelitis of the metatarsal head and/or proximal phalanx, septic arthritis of the metatarsophalangeal joint (MTPJ), and gross infection of the toe. This method of amputation also provides an option of ensuring an adequate surgical debridement of the infected margins [8].

The ankle brachial index (ABI) ≥ 0.8 and the toe brachial index (TBI) ≥ 0.7 also suggest inclusion criteria for indications of a RA, however, due to the unavailability of a hand-held Doppler, these indexes could not be calculated. In the case of this patient who presented with paresthesia, pulselessness, and pallor of distal extremities, calculation of these indexes (ABI and TBI) would have also aided in confirming the differential diagnosis of PAD as an ABI value below 0.9 is diagnostic of PAD. As stated above, an inclusion ABI range of 0.8 and above, even if proposed as an inclusion criterion for a RA, is also indicative of moderate arterial disease (ABI: 0.5-0.8) and requires a referral to a Vascular specialist who could deem the need for limb revascularization if necessary.

However, in patients with diabetic foot who do not undergo or are not suitable candidates for revascularization, and are treated with a metatarsal amputation (e.g., RA), the ABI alongside serum albumin are significant predictors of wound healing post-amputation. An ABI range of 0.7-1.3 improves wound healing rates from 80% to 100% whereas a serum albumin level of ≥ 30 g/L shows wound healing rates up to 70% [8]. Therefore, serum albumin is a contributing factor to a patient’s wound healing outcomes as an albumin deficiency can prolong the inflammatory phase (with acute phase reactants) in wound inflammation, causing reduced fibroblast production and a lack of collagen biosynthesis inhibiting the neoangiogenesis process. This deficiency can be corrected by albumin infusions and a boost in dietary protein intake as increased physiological demands causing malnutritional status are highly prevalent in diabetic patients [9]. In the case of this patient, he maintained a serum albumin range of 23-25 g/L (laboratory reference range: 35-55 g/L) throughout the course of his hospital stay with no specific interventions taken as it was prioritized to firstly control his blood glucose level which was only completely controlled by post-operative day 15.

There are various other factors that influence wound healing that can be classified into three categories: the eradication of pathogenic bacteria and biofilm, maintenance of excellent blood supply and proper oxygenation, and lastly, the presence of necessary immunological healing factors [10].

In the eradication of pathogenic bacteria, as prevalent in this patient’s wound swab culture, this was treated with IV broad-spectrum antibiotics (Flagyl and Augmentin) and mechanical debridement with daily foot soaks in a vinegar-based bath. Given the nature of the wound and surrounding polymicrobial infection, broad-spectrum antibiotics such as Augmentin and Flagyl are chosen because they have Gram-positive, Gram-negative, and anerobic coverage [11]. These antibiotics were administered for a three-week period due to the severity of the infection and discontinued the day prior to discharge of the patient. The goal of mechanical debridement is to maintain a moist environment by dressing the wound with wet gauze and changing them on a daily basis which is optimum for the healing process.

Vinegar (acetic acid) is an inexpensive, readily available, and non-toxic alternative to many known widely used topical agents such as povidone-iodine, hydrogen peroxide, and sodium hypochlorite which can be cytotoxic in nature causing adverse effects in the wound healing process. Acetic acid is known to be efficacious against an extensive range of organisms, inclusive of the isolated organisms on the patient’s wound culture namely Pseudomonas Aeruginosa and Streptococcus. These organisms need a pH environment of ≥ 6 to survive, therefore, acetic acid reduces the pH of the wound thereby inhibiting any further growth of the mentioned organisms. A lower wound pH also contributes to improved cellular oxygenation by the Bohr Effect and increases oxygen radical production thereby eradicating these bacteria [11]. However, for optimal results of rapid decontamination and improved granulation in a two-week period, treatment must be carried out with gauze soaked in 1% acetic acid. The pH of the immersion baths can be 0.1% acetic acid to create an acidic environment and must be measured accurately with a pH meter and only diluted with 0.9% normal saline [12]. In the case of this patient, a uniform layer of pink granulation tissue was seen during week 4 (Day 22) of post-operative treatment which is indicative of the success of the treatment protocol put in place.

However, ischemia in his Great Left toe caused a complication during his post-operative care, necessitating another RA on post-operative day 10 after it was first observed on post-operative day 5. Despite the decision to initially observe the toe in the hopes of preserving the digit, the toe already had necrotic tissue on its plantar surface, as shown in Figure 1. This necrotic tissue could have inhibited blood flow and an adequate oxygen supply, resulting in toe ischemia. This complication could have been avoided if the patient's first surgery included debridement and a RA of the great left toe alongside the second left toe.

As a long-term diabetic patient with uncontrolled diabetes and newly diagnosed atrial flutter which can slightly increase his risk of embolization, he is at an increased risk of PAD. He was given Carvedilol 3.25 g as it has shown to increase insulin sensitivity in both hypertensive and diabetic patients [13] and as a preventative measure to reduce his risks of mortality, as cardiovascular disease is the leading cause of death in diabetics. 

## Conclusions

Diabetic foot ulcers in patients with long-standing DM are a chronic wound condition. Diabetic patients with DFUs who receive the recommended treatment for their specific condition require a well-planned multi-disciplinary approach in their management, including blood glucose control, treatment of other underlying comorbidities, wound care plan, and foot-care plan with special footwear modifications, medical nutrition therapy, physiotherapy, and/or rehabilitation. 

Healthcare workers in multi-disciplinary teams need to work together to create better awareness of diabetic complications and provide continued counseling for diabetes self-management education to patients and their families. This is done to encourage treatment adherence and prevent delaying routine health checkups.

Due to the high prevalence of DM in Jamaica and the recurring presentation of DFUs in regional hospitals with a DM diagnosis also encountered as an incidental finding in both younger and older populations, a recommendation is made to medical departments to incorporate a Scoring Metric or Classification system that is tested and deemed applicable to nation-wide hospitals to guide treatment of these types of wounds.
